# HPLC-Based Chemometric Analysis for Coffee Adulteration

**DOI:** 10.3390/foods9070880

**Published:** 2020-07-04

**Authors:** Wai Lok Cheah, Mingchih Fang

**Affiliations:** Department of Food Science, College of Life Science, National Taiwan Ocean University, No 2, Beining Rd., Keelung City 20224, Taiwan; wailokc@yahoo.com

**Keywords:** coffee, adulteration, chemometrics, PCA, food fraud

## Abstract

Coffee is one of the top ten most adulterated foods. Coffee adulterations are mainly performed by mixing other low-value materials into coffee beans after roasting and grinding, such as spent coffee grounds, maize, soybeans and other grain products. The detection of adulterated coffee by high performance liquid chromatography (HPLC) is recognized as a targeted analytical method, which carbohydrates and other phenolic compounds are usually used as markers. However, the accurate qualitation and quantitation of HPLC analyses are time consuming. This study developed a chemometric analysis or called non-targeted analysis for coffee adulteration. The HPLC chromatograms were obtained by direct injection of liquid coffee into HPLC without sample preparation and the identification of target analytes. The distinction between coffee and adulterated coffee was achieved by statistical method. The HPLC-based chemometric provided more characteristic information (separated compounds) compared to photospectroscopy chemometric which only provide information of functional groups. In this study, green Arabica coffee beans, soybeans and green mung beans were roasted in industrial coffee bean roaster and then ground. Spent coffee ground was dried. Coffee and adulterants were mixed at different ratio before conducting HPLC analysis. Principal component analysis (PCA) toward HPLC data (retention time and peak intensity) was able to separate coffee from adulterated coffee. The detection limit of this method was 5%. Two models were built based on PCA data as well. The first model was used to differentiate coffee sample from adulterated coffee. The second model was designed to identify the specific adulterants mixed in the adulterated coffee. Various parameters such as sensitivity (SE), specificity (SP), reliability rate (RLR), positive likelihood (+LR) and negative likelihood (−LR) were applied to evaluate the performances of the designed models. The results showed that PCA-based models were able to discriminate pure coffee from adulterated sample (coffee beans adulterated with 5%–60% of soybeans, green mung beans or spent coffee grounds). The SE, SP, RLR, +LR and −LR for the first model were 0.875, 0.938, 0.813, 14.1 and 0.133, respectively. In the second model, it can correctly distinguish the adulterated coffee from the pure coffee. However, it had only about a 30% chance to correctly determine the specific adulterant out of three designed adulterants mixed into coffee. The SE, RLR and −LR were 0.333, 0.333 and 0.667, respectively, for the second model. Therefore, HPLC-based chemometric analysis was able to detect coffee adulteration. It was very reliable on the discrimination of coffee from adulterated coffee. However, it may need more work to tell discern which kind adulterant in the adulterated coffee.

## 1. Introduction

The coffee bean is one of the most widely traded agricultural commodities and its consumption has increased rapidly [1]. Many gourmets say what you choose to drink in the morning tells what kind of person you are. The methods of coffee roasting, brewing and drinking are currently hyped up to an art in certain specialty coffees. Animal-passed coffee beans may be one of the most poetic examples. Based on the Database of United States Pharmacopeia Convention (USP) [2], coffee is the top ten adulterated food products due to its high commercial value and the shortage of coffee beans worldwide. Coffee adulteration may be performed by changing the quality of beans or adding other low-cost coffee and non-coffee materials, such as spent coffee grounds, corn, barley, maize, soybeans and other grains which bear a resemblance to coffee beans in term of color, particle size and texture [3,4]. Roasted soybeans have been found to be a good adulterant of coffee, because their color, flavor and aroma are similar to coffee. The making process has even be patented [5]. The contamination of low-priced robusta species in claimed 100% Arabica coffees is another means of coffee fraud. There are also cases of coffee products being recalled due to presence of undeclared drug contents, namely, sildenafil and tadalafil [6]. In order to protect consumers and ensure food safety, the authenticity assessment of coffee products is a positive way of approaching the problem of coffee fraud.

Several analytical methods have been proposed to identify the adulteration of ground roasted coffee, including gas chromatography-mass spectrometry (GC-MS) [3], high-performance liquid chromatography (HPLC) [7,8,9], ultraviolet-visible spectrophotometry (UV-Vis) [10], ultra-performance liquid chromatography (UPLC) [11], Fourier-transform infrared spectroscopy (FTIR) [12] and voltammetric electronic tongue [13]. The above methods apply either targeted- or non-targeted analysis methods. Targeted analysis methods are broadly used to detect food contaminations—as well adulterations—which focus on detecting one or more compounds in a specimen. Specific targeted methods are generally complicated but can detect analytes in part per trillion (ppt) levels in complex matrices [14]. However, most of the adulterations are unknown additive compounds, so the targeted analysis is not always effective. Non-targeted methods such as chemometric analyses are the combination of emerging analytical methods and statistic software to detect food adulterations [15]. Non-targeted analysis treats food data as fingerprints. These indicate authenticity and provide early warning information for adulterated food. Therefore, the development of an HPLC-based non-targeted analysis to detect coffee adulteration is paramount.

In a review of the literature, detecting coffee adulteration by using liquid chromatography belongs to a target analysis method that analyzes compounds (carbohydrates) in coffee and adulterated coffee to accomplish the distinction [7,8,9,11]. In contrast, spectroscopy methods have evolved as non-target analytical methods to detect coffee adulteration. For example, one study used ultraviolet-visible (UV-Vis) spectroscopy and successive projection algorithms (SPA) to construct a linear discriminant analysis (LDA) model for the identification of adulterants in ground roasted coffee [10]. In many coffee researches, coffee beans were roasted in convection ovens in laboratories to the desired color and intensity. The heating/roasting effect of a conventional oven was slow and inhomogeneous. Hence, the roasting time was long—40 min on average for a batch [12,16], while a commercial coffee bean roaster only requires 12–15 min. In order to simulate the commercial roasting method and enhance the representativeness of the sampling, industrial roaster was used in the study. In addition, many chemometric spectroscopy methods for coffee adulteration have been conducted on the powdered form of coffee [12], or extracted by solvents such as CDCl_3_ for nuclear magnetic resonance (NMR) analysis [17]. However, coffee is normally consumed in liquid form, and hot water is the only solvent. In this study, commercial roasting and brewing methods were applied for coffee sample preparation. Non-targeted analysis, coupled with HPLC was developed for the discrimination among roasted coffee, common adulterants and mixtures of coffee and adulterants. The statistical method used in this research was principal component analysis (PCA). Two models were developed in order to evaluate the ability of the method to detect coffee adulteration, as well as to identify the adulterants in the adulterated sample.

## 2. Materials and Methods

### 2.1. Coffee and Adulterants

Green Arabica coffee beans (Chanchamayo, Peru) were purchased from Ho Hsin Beans Trading Co., Ltd (Taichung, Taiwan). Soybeans and green mung beans were obtained from a local supermarket. Spent coffee grounds were supplied by chain 85 °C Bakery Cafe shop in Taiwan and kept frozen (−12 °C) until use.

### 2.2. Roasting

**Figure 1:**
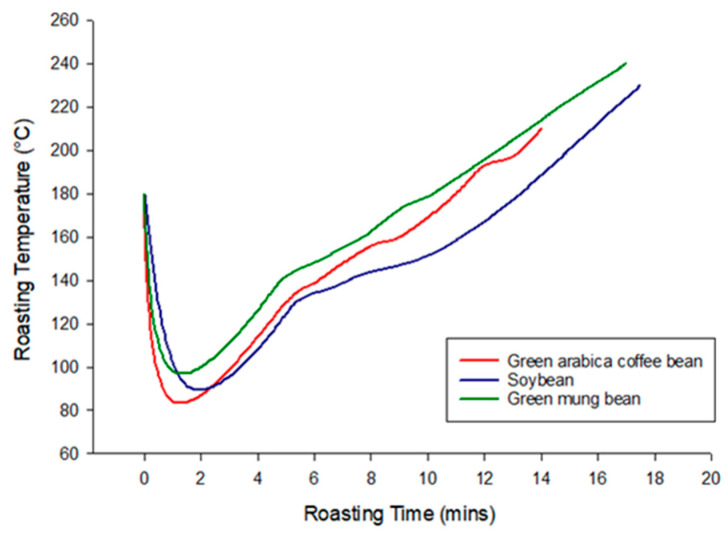
Roasting curves of green Arabica coffee beans, soybeans and green mung beans.

Spent coffee grounds were defrosted at room temperature for 18 h, and then dried in a convection oven (Model DK-500DT, Bioman, New Taipei, Taiwan) at 100 °C for 5 h. In order to simulate the roasting methods of commercial coffee in this study, an industrial-grade 800-N coffee bean roaster (Yang Chia Machine Works, Taichung, Taiwan) was used. Coffee beans (500 g), soybeans (500 g) and green mung beans (500 g) were each subjected to the coffee bean roaster at 180 °C. The temperature went down to the turning point and went up to 160 °C in 8 min, which was the dehydration process of coffee beans and adulterants. The first cracking sound happened in the coffee beans at 11.5 min and 195 °C. The other materials did not crackle. Coffee beans, soybeans and green mung beans were removed to an air-cooling chamber when the temperature rose to 210 °C, 230 °C and 240 °C, respectively. The roasting times were also different for each sample: 11–14 min for roasting coffee beans, 16–18 min for roasting the soybeans and 16–17 min for roasting the green mung beans. The roasting end-point was determined by the appearance in color to the desired brown. The roasting curves of coffee beans, soybeans and green mung beans are shown in Figure 1. Samples were grinded by electric grinder (Model 600, Yang Chia Machine Works, Taichung, Taiwan) after roasting and submitted to color evaluation.

### 2.3. Color Evaluation

A tristimulus colorimeter (TC-1800 MK-II, Tokyo Denshoku Co., Tokyo, Japan) was used to measure the luminosity (CIELAB L*) of the samples, which was the most relevant parameter above a* and b* for roasted coffee beans [18]. It was also successfully employed as a reference for roasting degree [16]. Comparing the value of luminosity, the degree of coffee-roasting can be classified into three groups: light (23.5 < L* < 25.0), medium (21.0 < L* < 23.5) and dark (19.0 < L* < 21.0) [16]. The measurement of luminosity was performed in three replicates.

### 2.4. Experimental Design

Coffee beans, soybeans and green mung beans were each from three roasting batches (for three replicates). Spent coffee grounds were obtained from three different dates. Ten pure materials of each batch including coffee beans, soybeans, green mung beans and spent coffee grounds were prepared as pure samples—in total 4 × 10 × 3 = 120 samples (4 materials, 10 replicates, 3 batches). The adulterated samples were prepared by intentionally mixing coffee beans and adulterants at different ratios (coffee: adulterants, 95:5, 90:10, 80:20, 60:40, 40:60% *w/w*) in every batch (3 × 3 × 5 = 45, 3 batches, 3 adulterants, 5 concentrations). Cross-batch mixtures were also prepared (3 × 5 = 15, 3 adulterant cross-batches, 5 concentrations). In total, 60 adulterated samples were prepared. All 180 samples were submitted to HPLC analysis.

### 2.5. Chromatograms Acquisition of Brewed Coffee and Adulterated Coffee

A Shimadzu LC-2040C Plus (Shimadzu Co., Kyoto, Japan) equipped with an Agilent Zorbax SB-Phenyl C18 column (150 mm × 4.6 mm, 5 µm) and a PDA (photodiode-array) detector was used in the measurement. The mobile phases were pure water (solvent A) and methanol (solvent B). The gradient elution was operated as flowing: 0–4 min: 2% B, 4–8 min: 4%–10% B, 8–13 min: 10%–20% B, 13–20 min: 20%–35% B, 20–27 min: 35%–90% B, 27–27.5 min: 90%–2% B, 27.5–30 min: 2% B. The injection volume and flow rate were 20 µL and 1 mL min^−1^, respectively. The PDA was set at scan range between 200 and 800 nm; the chromatogram was monitored at 254 nm.

Coffee and adulterated coffee samples were well-brewed in order to simulate commercially brewed coffee. The water temperature was controlled in the range of 91–94 °C. Two grams each of ground coffee beans and adulterated coffee samples were brewed with 25 mL water for 10 min. Each sample was centrifuged at 1000 × g for 5 min and then filtered with No. 1 filter paper. The filtrate was passed through a 0.2-μm PTFE filter and then transferred into a 1.5 mL vial for analysis.

### 2.6. Statistical Analysis

SPSS version 22 (Statistical Product and Service Solutions, IBM Co., New York, NY, USA) was used to evaluate the color measurements between the samples of coffee and adulterated coffee in order to ensure uniformities among batches, adulterants and mixtures. An independent *t*-test was used in this analysis. Principle component analysis (PCA) with normalized HPLC chromatogram (retention time alignment of peaks) data was applied to distinguish adulterated coffee from roasted coffee samples. For PCA analysis, data metrices were constructed (each row corresponded to a sample; each column represented peak intensity at a given retention time) and handled by MATLAB software version 2018a (MathWorks, Natick, MA, USA) with the Classification Learner software application. Two models were built to us the PCA data. The first model was built with 180 samples (30 for pure coffee, 30 for pure soybeans, 30 for pure green mung beans, 30 for pure spent coffee grounds, and 60 for adulterated coffee) aimed on the authentication of coffee samples. The second model was built with 150 samples (180 samples from the first model, excluding 30 samples of pure coffee) to test the ability to identify the adulterants. In the model experiments, 60% of the samples were randomly selected as training set; the others were treated as the test set.

The performances of the studied models were characterized by evaluating the quality of figure of merit (FOM). Figure of merit correspond to numeric parameters such as specificity (SP), sensitivity (SE) and reliability rate (RLR) [19]. The definition of the specificity (SP) was the ratio of true-negative samples (TN) to the sum of false-positive samples (FP) and the total number of known-negative samples (equation 1). It provided a measure of how well the model can predict samples of the class of controls. The sensitivity (SE) defined as the ratio of true positive samples (TP) to the sum of false-negative samples (FN) and the total number of known positive samples (equation 2). It provided a measure of how well the model correctly identify samples of a given class [20]. The reliability rate (RLR) provided an overview of the trueness of the model and its definition was shown in equation 3 [12].

(1) $$\mathrm{Specificity}\;\left(\mathrm{SP}\right)=\frac{TN}{\left(TN+FP\right)}$$

(2) $$\mathrm{Sensitivity}\;\left(\mathrm{SE}\right)=\frac{TP}{\left(TP+FN\right)}$$

(3) $$\mathrm{RLR}=1-\left(\left(\frac{FN}{TP+FN}\right)+\left(\frac{FP}{TN+FP}\right)\right)=\mathrm{SP}+\mathrm{SE}-1$$

In the first model, the TP was defined as the adulterated sample and was successfully detected by the system. The TN was the pure coffee sample or other pure adulterants and was successfully detected. The FP was the pure coffee sample but was misclassified as adulterated sample. The FN was the adulterated sample but was misclassified as pure coffee or other adulterants. In the second model, the TN was the pure adulterant and successfully detected. The FP was the pure adulterant and misclassified as adulterated coffee. TP was defined as the adulterated sample and the specific adulterant was successfully identified. The FN was the specific adulterant in the adulterated coffee which was classified as the other adulterant in the adulterated coffee or the pure adulterant.

The positive likelihood ratio (+LR) and the negative likelihood ratio (−LR) were also applied to evaluate the performance of the studied models. The definitions of +LR and −LR are listed in equation 4 and equation 5 [21]. A bigger value of +LR gave the stronger confidence of the positive result, while a smaller value of −LR presented the higher value of the testing results [22].

(4) $$+\mathrm{LR}=\frac{SE}{1-SP}$$

(5) $$-\mathrm{LR}=\frac{1-SE}{SP}$$

## 3. Results and Discussion

### 3.1. Coffee Roasting and Color Measurement

The color measurement was performed to evaluate whether the appearance of the roasted adulterants and the coffee were similar after roasting and grinding. Table 1 shows the L* value of roasted coffee (ground) was significantly different from roasted soybeans, green mung beans and spent coffee grounds (*p* < 0.05) and spent coffee grounds was the darkest one (L* was the lowest 18.0). Similar studies of roasted coffees and adulterants in convection ovens with precisely controlled temperatures, such as 240 °C 11 min for coffee, 240 °C 30 min for corn and 250 °C 28 min for barley resulted in color similarity [16]. In this study, soybeans and green mung beans required higher roasting temperatures and time to reach the desired color. The industrial-grade coffee bean roaster continually increased the temperature over time during the roasting and it was not easy to control, compared to convection oven-roasting in which there is a constant temperature during the whole roasting procedure. We tried to roast the adulterants at higher temperatures and for longer times. In these overheated roasting conditions, a similar color was obtained, but the flavor was burnt. Therefore, we decided to roast the adulterants in coffee-bean way. The final temperature was slightly increased to 230 °C and 240 °C for soybeans and green mung beans, respectively. The maximum temperature of the commercial roaster for coffee was 240 °C. The roasting time for adulterants was extended to around 18 min as shown in Figure 1. The condition resulted in good-smelling soybeans and green mung beans; the soybean especially smelled like coffee. Though the color of adulterants and coffee was different, there was no significant difference among all adulterated coffees in which coffee was mixed with 20% adulterants (*p* > 0.05).

### 3.2. HPLC Chromatograms

**Figure 2:**
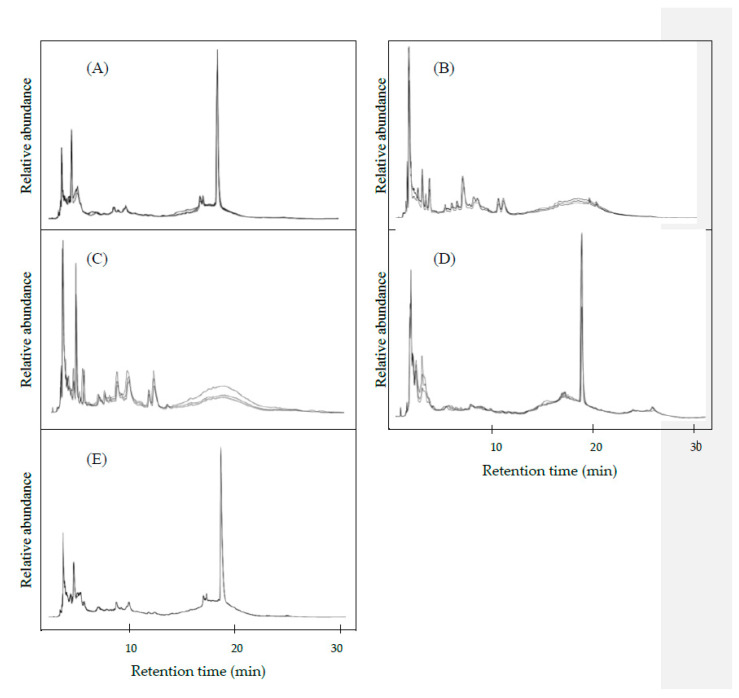
HPLC chromatograms (overlap of three) of (**A**) coffee beans, (**B**) soybeans, (**C**) green mung beans, (**D**) spent coffee grounds and (**E**) adulterated coffee beans containing 20% soybeans.

Applications of HPLC for the detection of adulterated coffee were mainly based on targeted methods. Analytes such as oligosaccharides, monosaccharides, trigonelline, and/or nicotinic acid were applied as markers to identify adulterants in coffee [7,8,9,11]. In this study, HPLC with non-targeted analysis was developed to differentiate coffee adulteration. HPLC analysis was directly performed after sample extraction and the generated chromatogram was utilized as a “fingerprint”. Figure 2 shows the HPLC chromatograms of pure coffee, pure adulterants and adulterated coffee containing 20% soybeans. Coffee beans (Figure 2A), soybeans (Figure 2B) and green mung beans (Figure 2C) showed very distinct chromatograms because each material presented their own special compounds, such as caffeine, tannic acid, linoleic acid, nicotinic acid, chlorogenic acids and trigonelline in coffee [23]. Soybeans contained isoflavones [24]. Green mung beans contained polyphenols, polysaccharides and peptides [25]. However, the chromatograms of coffee, spent coffee grounds (Figure 2D) and adulterated coffee beans (Figure 2E) showed high similarities. Chemometric approaches were employed to discriminate chromatograms generated from different adulterants and adulterated coffee beans.

### 3.3. Principal Component Analysis (PCA)

**Figure 3:**
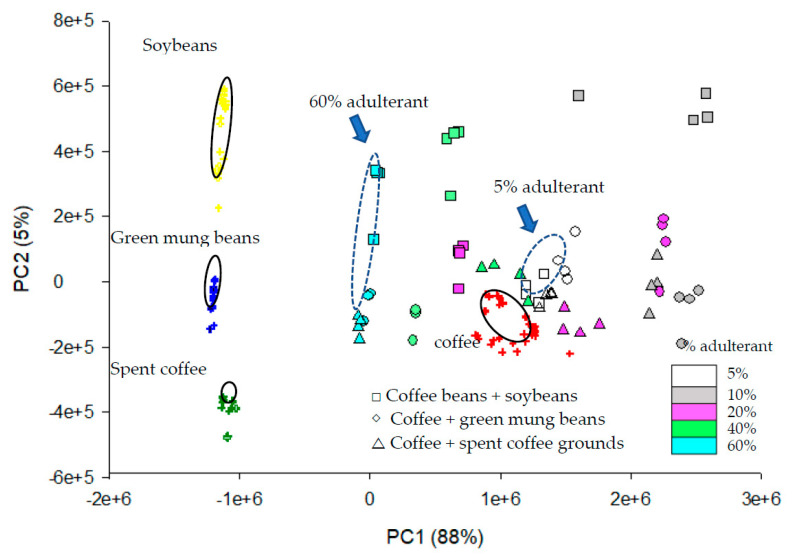
PCA-scores scatter plot of coffee beans in comparison to soybeans, green mung beans, spent coffee grounds and adulterated coffee containing different adulterants in different mixing ratios (5%, 10%, 20%, 40% and 60%).

The PCA-scores scatter plot derived from normalized HPLC chromatograms is shown in Figure 3. Pure coffee, soybeans, green mung beans and spent coffee grounds are well-separated. The coffee sample was more to the positive of PC1 and the others were more to negative of PC1. The successful separation among coffee, soybean and green mung bean was as expected, because each material contained different compounds and presented a specific chromatogram. The separation between coffee and spent coffee grounds was very good as well. Detailed elements from PCA analysis indicated that some small peaks appeared between 20–25 min in HPLC chromatograms of spent coffee grounds (Figure S1) contributed most to the discrimination between spent coffee grounds and coffee.

**Figure 4:**
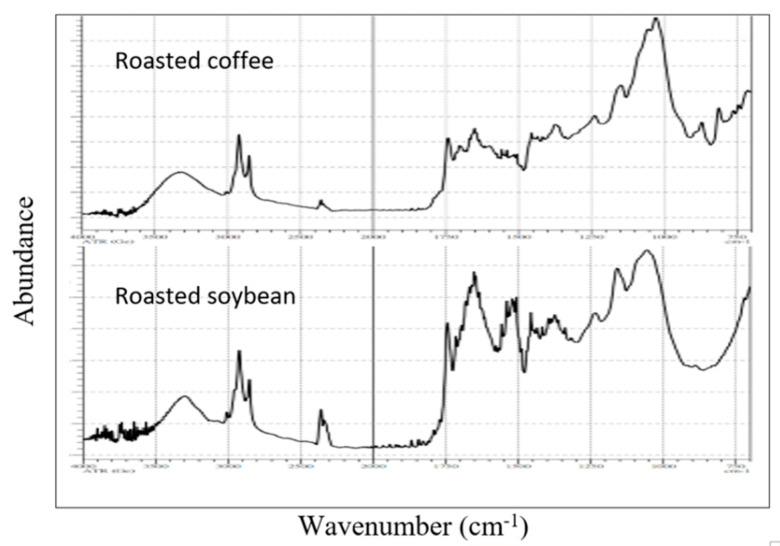
FTIR (ATR, Ge) spectra of roasted coffee beans and soybeans.

The PCA values of adulterated coffees were disorderly around pure coffee. The 60% adulterated coffee obtained values close to zero in PC1 and close to adulterants as well. The various mixing ratios (5%, 10%, 20%, 40% and 60%) are displayed in different colors in Figure 3. The results indicated that it was possible to detect coffee adulteration with various adulterants in admixture as low as 5% (*w/w*). This study was able to use HPLC chromatograms for chemometric analysis. However, in Figure 2, it is clear that overlapped HPLC chromatograms did not perfectly match each other. In many chemometric applications, the spectra—especially the most adopt FTIR spectra—are almost identical to each other in the same test group. The uses of HPLC chromatograms as fingerprints for chemometric analysis is powerful because HPLC chromatograms provide more distinct and detailed information, due to the peaks represented to different compounds. While using FTIR for chemometric analysis, the peaks only represent different functional groups. Figure 4 shows the FTIR spectra of roasted coffee beans and soybeans. They were similar in certain degree; compared to Figure 2A,B the chromatograms of coffee and soybean were very different. However, the system stability and chromatogram reproducibility of HPLC was not as good as spectrophotometric method (e.g., FTIR). In this study, the detection limit of adulterants in coffee was set at 5%. Compared to many chemometric analyses with IR spectra, the detection limit was below 1% [26].

### 3.4. Discriminatory Power of Models Built

Two models were built in this study. The first model was designed to distinguish pure coffee form adulterated coffee. The second model was designed to identify the adulterants that exist in the adulterated coffee. A total of 180 sample sets were divided into two groups including a training set (60% data) and a test set (40% of data). We used the training data set to calculate the parameters and to generate the models for the first and second models. The test set was used to test the classification results of the models. A confusion matrix was used to illustrate the test results.

**Figure 5:**
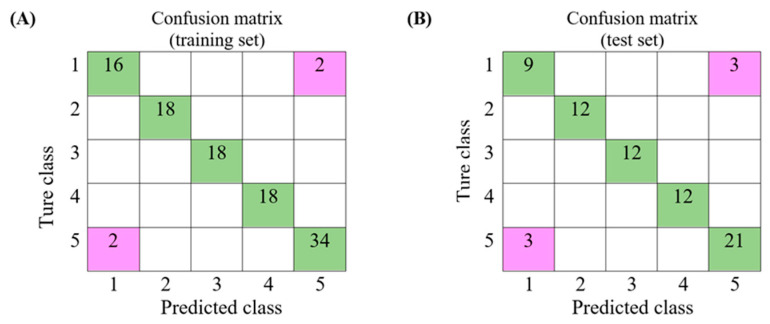
Confusion matrix of the first model that used to discriminate coffee from adulterated coffee for (**A**) training set and (**B**) test set (class 1—coffee; class 2—soybeans; class 3—green mung beans; class 4—spent coffee grounds; class 5—adulterated coffee beans).

In the first model, the classes 1–5 were coffee, soybean, green mung bean, spent coffee ground and adulterated coffee. Of 18 pure coffee samples (true class 1), 2 samples were classified as adulterated coffee. Of 36 adulterated coffees (true class 5), 2 samples were classified as pure coffee. The training set contained 2 false-positive (FP) samples (classifying pure coffee as adulterated coffee), 70 true negative (TN) samples (classifying unadulterated sample correctly), 2 false-negative (FN) samples (classifying adulterated coffee as pure coffee) and 34 true positive (TP) samples (classifying adulterated coffee correctly) (Figure 5A). These parameters were used to calculate the discriminatory power and are summarized in Table 2. Then, data from the test set were applied to the established first model. The results are shown in Figure 5B. Three FP and three FN were obtained. The first model was able to correctly distinguish 21 adulterated samples from 24 adulterated coffees. However, 3 pure coffees were recognized as adulterated coffees, and 3 adulterated coffees were recognized as pure coffees.

**Figure 6:**
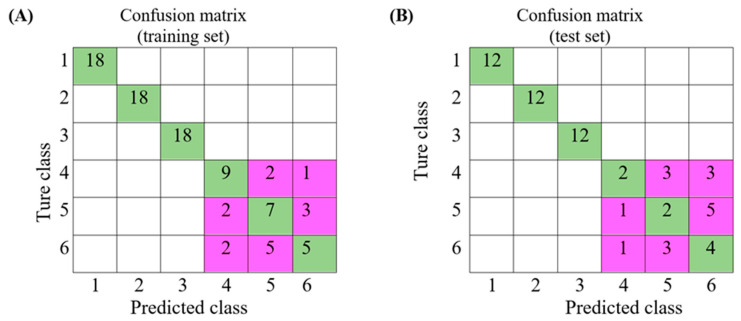
Confusion matrix of second model that used to identify the adulterants existed in the adulterated coffee for (**A**) training set and (**B**) test set (class 1—soybeans; class 2—green mung beans; class 3—spent coffee grounds; class 4—adulterated coffee containing soybeans; class 5—adulterated coffee containing green mung beans; class 6—adulterated coffee containing spent coffee grounds).

The second model was designed for distinguishing which kind of adulterants were in the adulterated coffees. The results are shown in Figure 6. In the training set, the pure soybeans, green mung beans and spent coffee grounds were correctly classified, while the adulterated coffees were partially classified. For example, in 12 soybean-adulterated coffees (class 4), 2 were classified as green-mung-bean-adulterated and one was classified as spent-coffee-grounds-adulterated (Figure 6A). In the test set of the second model, all 36 pure adulterants (12 for each) were distinguished the from adulterated coffee. However, the second model was not able to identify the adulterants correctly in the adulterated coffees. For example, in 8 green-mung-bean-adulterated coffees (Figure 6B, class 5), only 2 were correctly identified as green-mung-bean-adulterated, one was misclassified as soybean-adulterated and 5 were classified as spent-coffee-grounds-adulterated. In the second model, the FN samples were many and had a high chance of misjudging the adulterants existed in the adulterated coffee.

Figure of merit (FOM) was used to characterize the performances of the studied models. Statistic equations were applied (Equation 1–5) for the calculation of SP, SE, RLR, +LR and −LR. In first model, SP (0.938) and SE (0.875) of the test set were satisfied. The reliability rate (RLR) was 0.813 proved the trueness of first model [12]. Positive likelihood ratio (+LR) was used to evaluate whether the positive result was correctly tested. The high +LR value (>10) strongly indicated the positive result tested was correct [22]. The obtained +LR of the first model were 33.7 and 14.1 for training set and test set, respectively (Table 2). On the other hand, the −LR values were low (0.0576 for training set and 0.133 for test set) indicated there was strongly evidence to prove the TN result was detected correctly [22]. Thus, the performance of first model was good. In the second model, lower SE values were observed—0.583 and 0.333 for the training and test sets, respectively. The lower SE values indicated that the ability of the model to detect the positive sample was low. This model performed 100% in prediction of TN samples and 0 case of FP sample. The obtained SPs were 1.00 for either training or test set. Therefore, the RLRs of the second model were same as the SEs. However, the second model’s SP value was satisfied (means the second model detected the pure adulterants correctly), but the aim of this model was to identify the adulterant mixed in the adulterated coffee (positive sample). Therefore, the SE value was more important and relevant to the ability of the model. On the other hand, the −LR values were 0.417 and 0.667 for training set and test set, respectively, which represented no evidence to prove that the true negative result was correct [22]—even if the SP value was 1.0. This indicated that the −LR was better than the SP for explaining the performance of the model. Thus, the second model was only suitable for the discrimination of pure coffee and adulterated coffee, while the ability of identifying adulterants existed in the adulterated coffee was low.

## 4. Conclusions

HPLC-based chemometric analysis was performed and able to distinguish coffee from adulterated coffee with detection limit around 5%. This study simulated commercial roasting conditions in sample preparation and liquid sample was obtained by coffee brewing. The chemometric analysis applied chromatograms (information of compounds) instead of photospectra (information of functional groups) studied in most chemometric analysis. The experimental model was able to separate pure coffee and adulterated coffees. However, the performance of telling what kind of adulterants existed in the adulterated coffee still needs to be improved.

## Figures and Tables

**Table 1:** L* values of coffee beans, adulterants and adulterated coffee beans.

Analyte	Luminosity Measurement Result	
	L* (Mean ± SD, *n* = 3)	*p*-Value (*t*-Test of Coffee and Other Analytes)
Coffee beans	20.8 ± 0.8	
Soybeans	27.5 ± 0.4	0.00
Green mung beans	30.5 ± 1.2	0.00
Spent coffee grounds	18.0 ± 0.2	0.00
Coffee beans + soybeans *	21.4 ± 0.7	0.10
Coffee beans + green mung beans *	21.5 ± 0.6	0.07
Coffee beans + spent coffee grounds *	20.5 ± 0.4	0.28

* 20% roasted adulterant in coffee beans.

**Table 2:** Estimated figure of merit (FOM) of the first and second model.

	SP	SE	RLR	+LR	−LR
First model					
Training set	0.972	0.944	0.916	33.7	0.0576
Test set	0.938	0.875	0.813	14.1	0.133
Second model					
Training set	1.00	0.583	0.583	–	0.417
Test set	1.00	0.333	0.333	–	0.667

SP—specificity; SE—sensitivity; RLR—reliability rate; +LR—positive ratio likelihood; −LR—negative ratio likelihood.

## Supplementary Materials

The following are available online at https://www.mdpi.com/2304-8158/9/7/880/s1, Figure S1: HPLC chromatogram of coffee and spent coffee grounds sample for 20–25 min of retention time.
