# Supplementary figures and images for: HPLC-Based Chemometric Analysis for Coffee Adulteration
Source: Foods. 2020 Jul 4;9(7):880. doi: 10.3390/foods9070880 (PMC7404477; doi:10.3390/foods9070880)

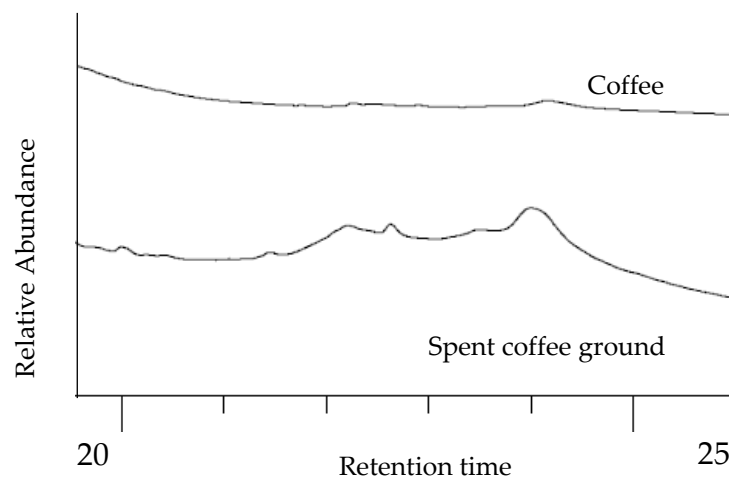

Figure S1. HPLC chromatogram of coffee and spent coffee ground sample for 20–25 mins of retention time.

Supplement: Supplementary file 1 [file foods-09-00880-s001.pdf]
